# Supplementary material for: Predicting microbial community responses to disturbance using genome-resolved trait-based life-history strategies
Source: ISME J. 2026 Apr 23;20(1):wrag099. doi: 10.1093/ismejo/wrag099 (PMC13196584; doi:10.1093/ismejo/wrag099)
Supplement: Supplementary-Material_wrag099 [file supplementary-material_wrag099.zip › Santillan_et_al_Sup_Info_wrag099.pdf]

Supplementary Information

Predicting microbial community responses to disturbance using genome-resolved trait-based life-history strategies

Ezequiel Santillan[1], Soheil Asgari Neshat[1], and Stefan Wuertz[1,2]*

[1]Singapore Centre for Environmental Life Sciences Engineering, Nanyang Technological University, Singapore, 637551, Singapore.

[2]School of Civil and Environmental Engineering, Nanyang Technological University, Singapore, 639798, Singapore.

*Correspondence to: Stefan Wuertz (swuertz@ntu.edu.sg)

Table S1. Community-level process performance data on day 42.

**Community function**[†]	**Disturbance frequency levels**[*]						**Welch's ANOVA**
	**0**	**1**	**2**	**3**	**4**	**5**	$\boldsymbol{P}_{\mathbf{BH}}$[§]
COD rem[¶] (%)	98.2 (1.3)	98.1 (0.7)	99.1 (0.7)	98.7 (0.4)	98.7 (0.2)	98.6 (0.3)	0.41
TKN rem[¶] (%)	95.5 (2.5)	97 (3.2)	97.3 (0.5)	97.8 (0.5)	96.1 (1.7)	54.6 (9.2)	**<0.001**
TKN	2.1 (1.1)	1.4 (1.5)	1.2 (0.2)	1.0 (0.2)	1.8 (0.8)	21.0 (4.3)	**<0.001**
NO_2^--N [mg/L]	3.7 (5.5)	3.9 (2.1)	0.8 (0.3)	2.2 (0.6)	2.8 (0.2)	0.0 (0.0)	**<0.001**
NO_3^--N [mg/L]	54.4 (6.1)	23.1 (8.1)	52.3 (2.7)	25.7 (3.7)	15.2 (1.1)	0.0 (0.0)	**<0.001**
PO_4^{3-}-P [g/L]	8.3 (0.4)	7.3 (0.5)	9.0 (0.4)	7.8 (0.5)	5.8 (0.5)	10.1 (0.3)	**<0.001**
SO_4^{2-}-S [g/L]	26.4 (0.3)	26.7 (1.0)	26.4 (0.3)	27.8 (1.7)	28.0 (1.8)	28.0 (2.2)	0.26
TA	36.4 (13.2)	177 (28.5)	54 (9.5)	173 (15.2)	224 (3.00)	468 (16.9)	**<0.001**
SVI	56.4 (7.2)	45.4 (6.9)	39.8 (4.7)	37.6 (4.0)	39.2 (4.5)	43.4 (2.5)	**0.017**
VSS:TSS (%)	88.6 (2.9)	94.4 (4.2)	93.6 (1.7)	91.6 (2.3)	92.6 (0.9)	93.8 (3.0)	0.16

[†] COD, chemical oxygen demand; TKN, total Kjeldahl nitrogen; TA, total alkalinity; SVI, sludge volume index; VSS:TSS, volatile to total suspended solids ratio.

[*] Average values (n = 5), with standard deviation in parentheses.

[¶] Removal of the indicated compound (percentage based on feed input).

[§] Welch's ANOVA test, Benjamini-Hochberg corrected *P* value (significant values in bold).

Table S2. Quality assessment of 133 medium- and high-quality metagenome-assembled genomes (MAGs) recovered from day-42 bioreactor metagenomes.

**Completeness**†	**Range**	**Count**	**Contamination***	**Range**	**Count**
moderate	50% - 69.9%	27	medium	5% - 9.9%	28
near	70% - 89.9%	49	low	0% - 4.9%	91
substantial	90% - 99.9%	55	none	0%	14
perfect	100%	2			

†Completeness categories based on the Minimum Information about Metagenome-Assembled Genomes (MIMAG) framework [38].

*Contamination categories and their corresponding counts.
